# Development and validation of a MODS risk prediction model for organophosphorus poisoning patients

**DOI:** 10.3389/fmed.2026.1791307

**Published:** 2026-05-12

**Authors:** Helong Yu, Ke Wang, Huisong Wu, Fangfang Li

**Affiliations:** Department of Emergency Medicine, Linquan County People’s Hospital, Fuyang, Anhui, China

**Keywords:** acute organophosphorus pesticide poisoning, blood lactic acid, endotracheal intubation, heart rate, multiple organ dysfunction syndrome, nomogram, prognosis

## Abstract

**Objective:**

Multiple organ dysfunction syndrome (MODS) is a major complication of patients with acute organophosphorus pesticide poisoning (AOPP) and is associated with high mortality. This study aimed to develop and validate a MODS prediction model for this patient population using a nomogram and machine learning methods.

**Methods:**

A retrospective study was conducted on 270 AOPP patients from Linquan County People’s Hospital to establish the prediction model. Lasso regression was used for variable selection, and multivariate Logistic regression was applied for model construction. Model performance was evaluated based on discriminative ability, calibration, and decision curve analysis.

**Results:**

Among the 270 AOPP patients, 129 (47.8%) developed MODS. The key predictors of MODS included heart rate, Endotracheal intubation, and blood lactic acid. The nomogram achieved an area under the curve (AUC) of 0.962 (95% confidence interval [CI]: 0.932–0.982). The calibration plot showed a high agreement between predicted probabilities and actual observed probabilities, and decision curve analysis demonstrated a favorable clinical net benefit of the model.

**Conclusion:**

We developed a risk prediction model for MODS in AOPP patients. This model can assist clinicians in assessing MODS risk and provide a scientific basis for subsequent interventions. External validation is required to confirm the reliability of the current risk model before its clinical application.

## 1 Introduction

Acute organophosphorus pesticide poisoning (AOPP) remains a major global public health concern, particularly in agricultural regions (1, 2). According to the World Health Organization (WHO), approximately 3 million people worldwide are affected by pesticide poisoning annually, with AOPP being the most prevalent form (3). Although effective antidotes are available for the clinical treatment of AOPP, patients with severe poisoning frequently progress to multiple organ dysfunction syndrome (MODS) (4, 5). MODS is defined as an acute and potentially reversible failure of two or more organ systems resulting from various pathogenic insults (6). Previous studies have demonstrated that organophosphorus compounds inhibit cholinesterase (ChE) activity, leading to substantial accumulation of acetylcholine in AOPP patients (7, 8). This triggers cholinergic system dysfunction, hypoxia, inadequate tissue perfusion, microcirculatory impairment, and disseminated intravascular coagulation (DIC), ultimately resulting in MODS (9). Despite continuous improvements in clinical management, the mortality of MODS remains high, exceeding 30% in critically ill patients (10), and is closely associated with the number and severity of impaired organs (11). Therefore, early identification and management of MODS risk factors are paramount for improving patient outcomes.

In recent years, predictive modeling has become an indispensable tool in acute poisoning care, supporting clinical decision-making, risk stratification, and resource allocation (12). Among various predictive tools, nomograms have been widely adopted due to their intuitive design, quantitative output, and ease of use in real-world clinical settings. Nomograms can individually estimate the probability of disease occurrence, clinical deterioration, or adverse outcomes by integrating multiple predictive variables into a visual scoring system (13). Their value in acute poisoning has been increasingly recognized: recent studies have successfully developed and validated nomograms for predicting adverse cardiovascular events in acute cardiotoxic poisoning, ICU admission in clozapine intoxication, and mechanical ventilation requirements in acutely intoxicated patients with impaired consciousness (12, 14, 15). These models have demonstrated favorable predictive performance and clinical practicability, highlighting that nomogram-based risk stratification can help physicians implement early, targeted interventions and improve prognosis in acute poisoning populations.

Concurrently, an effective and user-friendly predictive model was developed to assess the probability of MODS occurrence in AOPP patients. This aims to assist clinicians in providing appropriate management strategies and recommendations, thereby improving the long-term prognosis for AOPP patients.

## 2 Methods

### 2.1 Research subject

Patients diagnosed with AOPP at Linquan County People’s Hospital between 1 May 2022 and 31 September 2025 were selected. All patients received standardized treatment for acute organophosphorus pesticide poisoning immediately after admission. Gastric lavage and catharsis were performed as soon as possible for oral poisoning patients. Atropine was intravenously administered to achieve and maintain atropinization. Pralidoxime was used as a cholinesterase reactivator. Adjuvant treatments included fluid resuscitation, oxygen therapy, endotracheal intubation and mechanical ventilation for respiratory failure, circulatory support, anti-infective therapy, and correction of water–electrolyte and acid–base disturbances. Inclusion criteria: (1) All patients were coded with ICD-10 T60.0X1A (accidental) or T60.0X2A (self-harm) for acute organophosphorus pesticide poisoning; (2) met the diagnostic criteria for AOPP with definite exposure history, typical cholinergic symptoms, and decreased blood cholinesterase activity (16); and (3) Complete clinical documentation was available. The minimum sample size was calculated using the formula for two-group comparison in prediction model research:


$$n=\frac{{\left(Z_{1-\alpha /2}+Z_{1-\beta }\right)}^{2}\times [p_{1}(1-p_{1})+p_{0}(1-p_{0})]}{{\left(p_{1}-p_{0}\right)}^{2}}$$

With a type I error of 0.05, power of 0.90, *p*_1_ = 0.80, and *p*_0_ = 0.20, the minimum required sample size was 238 patients. Exclusion criteria: Patients meeting any of the following criteria were excluded: (1) Severe pre-poisoning organ dysfunction of the heart, liver, kidney, lung, or brain (e.g., heart failure, respiratory failure, liver failure, renal failure, or severe chronic neurological disease); (2) Concurrent hematological disorders or severe infections; and (3) Concurrent autoimmune diseases. This study was reviewed and approved by the Ethics Committee of Linquan County People’s Hospital (Approval No: SL-YJ2025-23).

### 2.2 Data collection

Baseline data: (1) Systematically collect baseline demographic characteristics and clinically relevant information for all enrolled patients, specifically covering age, gender, body mass index (BMI), comorbidities (such as coronary heart disease and diabetes mellitus), as well as blood pressure levels (BP), body temperature, respiration, heart rate (HR), and route of poisoning at the time of enrolment. (2) Laboratory parameters: Collected from enrolled patients included white blood cell count (WBC), hemoglobin (HGB), platelet count (PLT), serum albumin (ALB), globulin (GLOB), serum creatinine (Cr), urea nitrogen (Ur), serum cholinesterase (ChE), total bilirubin (TBIL), direct bilirubin (DBIL), indirect bilirubin (IBIL), alanine aminotransferase (ALT), aspartate transaminase (AST), alkaline phosphatase (ALP), serum bicarbonate (HCO3), blood glucose (GLU), blood lactate (Lac), amylase (AMY), creatine kinase (CK), C-reactive protein (CRP), and lactate dehydrogenase (LDH). All data were obtained during the initial assessment following the patient’s presentation and were verified and entered into the hospital electronic medical record system by two individuals.

### 2.3 Research design

This retrospective study defined the primary endpoint as the occurrence of MODS, characterized by a SOFA score of ≥2 points in two or more organ systems (17). Patients were grouped according to whether they developed MODS during treatment, and clinical baseline data were compared between the two groups.

### 2.4 Statistical analysis

This study employed SPSS 25.0, Stata 14.0, and R 4.1.3 software for statistical analysis. For continuous variables meeting the assumptions of normal distribution and homogeneity of variance, data were presented as $\bar{x}$ ± *s*. Comparisons between groups employed independent samples *t*-tests. Where data exhibited non-normal distribution or heteroscedasticity, they were expressed as median (M) (P25, P75), with intergroup differences analyzed using the Mann–Whitney U test. Categorical variables were expressed as frequencies and percentages, with pairwise comparisons performed using chi-square tests or Fisher’s exact probability tests. Missing values in all variables were below 10%, and all missing data were imputed using the sequential mean method. Lasso regression was employed to identify key risk and protective factors for MODS occurrence in AOPP patients, while simultaneously locking variables critical to MODS. Logistic regression was subsequently conducted to construct the final model. The predictive model’s discriminatory power and accuracy were assessed using the C-index and receiver operating characteristic (ROC) analysis. Furthermore, the model’s accuracy and clinical net benefit were validated through 1,000-resampled calibration curve and decision curve analysis (DCA). Finally, the robustness of the predictive model was further verified through internal validation. A two-tailed *p*-value < 0.05 was considered statistically significant.

## 3 Result

### 3.1 Inclusion of patients’ basic characteristics

A total of 300 patients diagnosed with AOPP were initially enrolled. After excluding 4 cases with severe infection, 8 cases with pre-existing heart failure, and 18 cases with incomplete clinical data, 270 patients were finally included in the analysis. The median age was 54 years, with 126 female patients (46.6%). Multiple organ injury was prevalent among AOPP patients, particularly involving the central nervous system (51.8%), lungs (40.7%), and cardiovascular system (31.1%), as detailed in Table 1. Among all patients, 66 had a history of hypertension (24.4%), 21 had a history of diabetes mellitus (7.7%), and 17 had a history of coronary heart disease (6.2%).

**Table 1:** Organ injuries in 270 patients undergoing AOPP.

Region	Number of cases	Percent (%)
Central nervous system	140	51.8
Lung	110	40.7
Cardiovascular system	84	31.1
Liver	82	30.3
Blood coagulation system	76	28.1
Pancreas	66	24.4
Kidney	30	11.1

AOPP, acute organophosphorus pesticide poisoning.

### 3.2 Comparison of demographic and laboratory parameters between the two patient groups

All laboratory parameters described in the Methods section are presented in Table 2. Among the 270 AOPP patients, 129 (47.8%) developed MODS during hospitalization. Patients in the MODS group were older, had shorter symptom durations, and needed tracheal intubation more often than those in the non-MODS group. Additionally, concurrent coronary heart disease was more common in the MODS group, although there were no statistically significant differences between the two groups in terms of gender, BMI, diabetes mellitus, or hypertension. Clinical indicators showed that patients in the MODS group had faster HR and significantly higher levels of WBC, ALT, AST, ALP, Cr, Ur, AMY, GLU, CK, CK-MB, LDH, CRP, Lac, and D-dimer levels compared to the non-MODS group (*p* < 0.05), while cholinesterase activity was significantly lower. At the same time, the MODS group showed delayed thrombin time (TT) and prothrombin time (PT) in coagulation function (*p* < 0.05). As shown in Table 2.

**Table 2:** Comparison of clinical baseline data between two groups of patients based on presence or absence of MODS.

Variables	Total (*n* = 270)	Non-MODS (*n* = 141)	MODS (*n* = 129)	*P*-value^a^^,^^b^^,^^c^
Age (year)	54 (40, 68)	47 (33, 58)	62 (52, 72)	<0.001*
Female [*n*(%)]	126 (46.6)	73 (51.8)	53 (41.1)	0.079
BMI (kg/m^2^)	25.35 (22.35, 28.09)	25.35 (22.35, 28.35)	24.89 (22.89, 27.89)	0.989
Delay time (h)	2 (1, 3)	2 (1, 4)	1.5 (1, 2)	<0.001*
Diabetes [*n*(%)]	21 (7.7)	7 (5.0)	14 (10.9)	0.071
Coronary heart disease [*n*(%)]	17 (6.2)	3 (2.1)	14 (10.9)	0.003*
Hypertension [*n*(%)]	66 (24.4)	31 (22.0)	35 (27.1)	0.326
Endotracheal intubation [*n*(%)]	110 (40.7)	2 (1.4)	108 (83.7)	<0.001*
Temperature (°C)	36.4 (36.2, 36.6)	36.4 (36.3, 36.6)	36.3 (36.1, 36.6)	0.030*
Breathing (times/min)	19 (16, 20)	19 (17, 20)	18 (15, 20)	0.010*
Heart rate (times/min)	99 (87, 110)	90 (83, 100)	110 (96, 123)	<0.001*
Systolic blood pressure (mmHg)	125 (108, 142)	126 (115, 138)	122 (97, 146)	0.107
Diastolic blood pressure (mmHg)	78 (68, 87)	81 (71, 87)	75 (60, 87)	0.006*
White blood cell count (×10^9^/L)	11.56 (8.16, 15.41)	9.44 (6.98, 12.67)	13.75 (10.74, 17.36)	<0.001*
Hemoglobin (g/L)	132.51 ± 20.09	130.18 ± 17.55	135.06 ± 22.33	0.046*
Platelet count (×10^9^/L)	228.50 (174.75, 272.25)	232.56 (189.00, 268.00)	223.00 (168.00, 275.00)	0.365
Cholinesterase (U/L)	397.00 (197.75, 1081.75)	806.00 (394.00, 2815.00)	225.00 (152.00, 397.00)	<0.001*
Total protein (g/L)	65.05 (60.00, 71.60)	65.10 (62.00, 70.50)	65.00 (58.00, 72.40)	0.426
Albumin (g/L)	39.80 (36.58, 43.40)	40.40 (37.50, 43.50)	39.30 (34.80, 43.10)	0.039*
Globulin (g/L)	25.10 (22.18, 29.13)	25.00 (23.00, 28.30)	25.30 (21.90, 30.20)	0.698
ALT (U/L)	21 (14, 30)	16.00 (12.00, 24.60)	22.50 (17.20, 37.00)	<0.001*
AST (U/L)	28 (20, 42)	23.00 (18.00, 29.00)	37.00 (26.00, 56.00)	<0.001*
Alkaline phosphatase (U/L)	83.00 (65.00, 100.25)	77.00 (60.00, 96.00)	90.00 (73.00, 104.00)	0.002*
Total bilirubin (μmol/L)	12.16 (8.65, 15.79)	11.90 (8.98, 15.31)	12.26 (8.23, 16.48)	0.961
Direct bilirubin (μmol/L)	2.88 (1.99, 4.39)	2.50 (1.78, 3.93)	3.50 (2.46, 4.78)	0.095
Indirect bilirubin (μmol/L)	9.40 ± 4.68	9.74 ± 4.19	9.03 ± 5.14	0.215
Serum creatinine (μmol/L)	60.05 (49.57, 74.08)	56.50 (47.64, 66.33)	65.00 (53.25, 85.80)	<0.001*
Blood urea nitrogen (mmol/L)	4.70 (3.70, 6.20)	4.40 (3.40, 5.20)	5.60 (4.20, 7.10)	<0.001*
Amylase (U/L)	113.00 (57.75, 197.59)	65.00 (47.00, 101.20)	197.59 (133.00, 283.00)	<0.001*
Blood glucose (mmol/L)	8.85 (5.90, 10.40)	6.30 (4.66, 8.44)	9.81 (9.81, 12.56)	<0.001*
Serum bicarbonate (mmol/L)	20.68 (19.90, 22.60)	21.10 (20.68, 24.20)	20.68 (16.90. 20.68)	<0.001*
CK (U/L)	150.00 (90.75, 210.57)	132.00 (83.00, 297.00)	180.00 (105.00, 218.00)	0.013
CKMB (U/L)	23.45 (13.48, 42.05)	16.00 (4.70, 24.30)	42.05 (24.00, 50.00)	<0.001*
LDH (U/L)	227.00 (187.00, 261.75)	196.00 (168.00, 236.00)	243.61 (226.00, 297.00)	<0.001*
CRP (mg/L)	0.50 (0.50, 7.99)	0.50 (0.50, 0.96)	1.12 (0.50, 16.17)	<0.001*
Lactic acid (mmol/L)	4.65 (1.88, 4.65)	3.20 (1.60, 4.65)	4.65 (2.70, 8.40)	<0.001*
APTT (s)	28.60 (25.38, 32.53)	29.40 (26.20, 32.40)	28.10 (23.60, 32.40)	0.081
PT (s)	13.70 (12.60, 14.70)	13.50 (12.70, 14.30)	13.90 (12.50, 15.40)	0.034*
TT (s)	17.20 (16.20, 18.30)	16.70 (16.00, 17.74)	17.74 (16.80, 19.30)	<0.001*
D-Dimer (μg/ml)	0.80 (0.34, 3.36)	0.44 (0.25, 1.10)	2.11 (0.68, 4.18)	<0.001*

a Continuous variables with normal distribution are presented as mean ± standard deviation (SD) and compared using independent-samples *t*-test.

b Non-normally distributed variables are presented as median (interquartile range, IQR) M(P25, P75) and compared using Mann–Whitney U test.

c Categorical variables are presented as *n* (%) and compared using χ^2^ test or Fisher’s exact test.

* The differences were considered to be statistically significant. MODS, multiple organ dysfunction syndrome; ALT, alanine aminotransferase; AST, aspartate aminotransferase; CK, creatine kinase; CKMB, creatine kinase-MB; LDH, lactate dehydrogenase; CRP, C reactive protein; APTT, activated partial thromboplastin time; PT, prothrombin time; TT, thrombin time.

### 3.3 Selection of predictors for MODS in patients with AOPP

**Figure 1:**
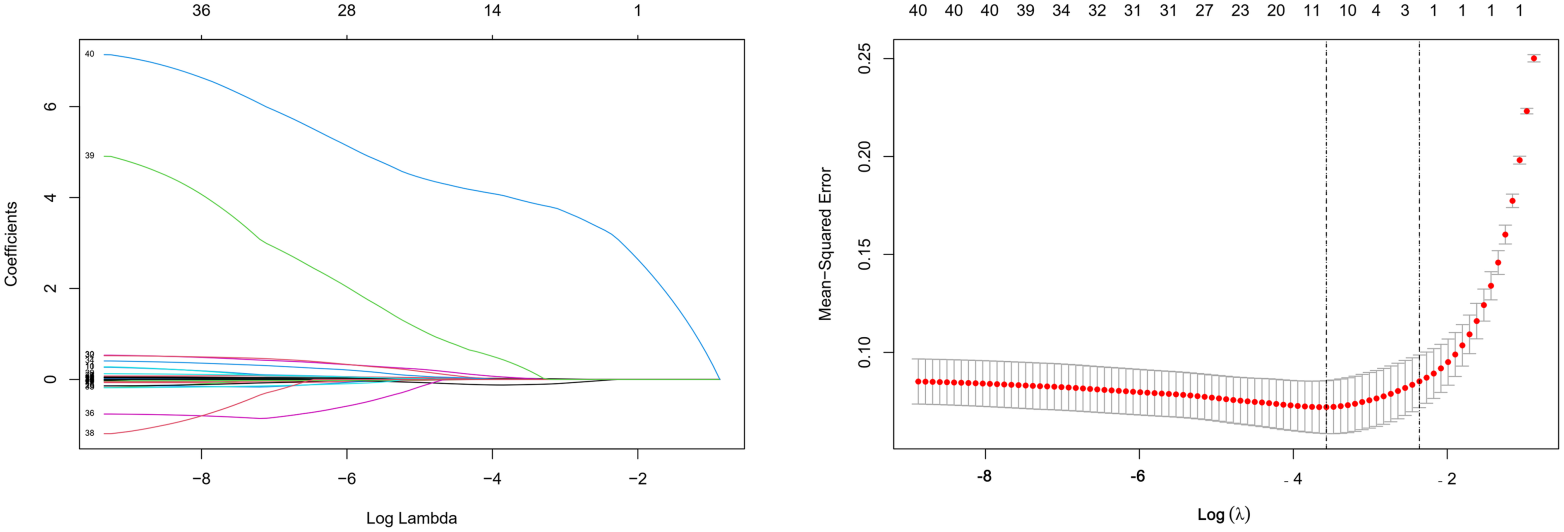
Screening of predictors using lasso regression analysis.

As seen in Figure 1, we used Lasso regression analysis to decrease the 40 patient variables to 14 based on non-zero coefficients. Multivariate logistic regression further identified three independent predictors for MODS in AOPP patients: heart rate (95% CI 1.012–1.078), Endotracheal intubation (95% CI 29.966–912.240), and lactate (95% CI 1.077–2.065), as shown in Table 3.

**Table 3:** Multivariate logistic regression analysis of MODS in AOPP patients.

Variables	Logistic regression model	
	OR (95% CI)	*P-*value^a^
Age (year)	1.025 (0.989 ~ 1.063)	0.170
Heart rate (times/min)	1.044 (1.012 ~ 1.078)	0.008*
Coronary heart disease [*n*(%)]	3.351 (0.385 ~ 29.167)	0.273
Endotracheal intubation [*n*(%)]	165.336 (29.966 ~ 912.240)	<0.001*
Cholinesterase (U/L)	1.000 (0.999 ~ 1.000)	0.070
Serum creatinine (μmol/L)	1.003 (0.969 ~ 1.038)	0.863
Amylase (U/L)	1.002 (0.999 ~ 1.005)	0.227
Alkaline phosphatase (U/L)	1.007 (0.9951.019)	0.247
Blood glucose (mmol/L)	1.046 (0.957 ~ 1.144)	0.318
LDH (U/L)	1.002 (0.993 ~ 1.011)	0.626
Lactic acid (mmol/L)	1.491 (1.077 ~ 2.065)	0.016*
Serum bicarbonate (mmol/L)	0.946 (0.785 ~ 1.140)	0.563
PT (s)	1.295 (0.882 ~ 1.902)	0.188
TT (s)	1.153 (0.721 ~ 1.845)	0.552

a Multivariate logistic regression analysis was used to determine independent risk factors.

*The differences were considered to be statistically significant. AOPP, acute organophosphorus pesticide poisoning; MODS, multiple organ dysfunction syndrome; LDH, lactate dehydrogenase; PT, prothrombin time; TT, thrombin time.

### 3.4 Constructing a risk prediction model

**Figure 2:**
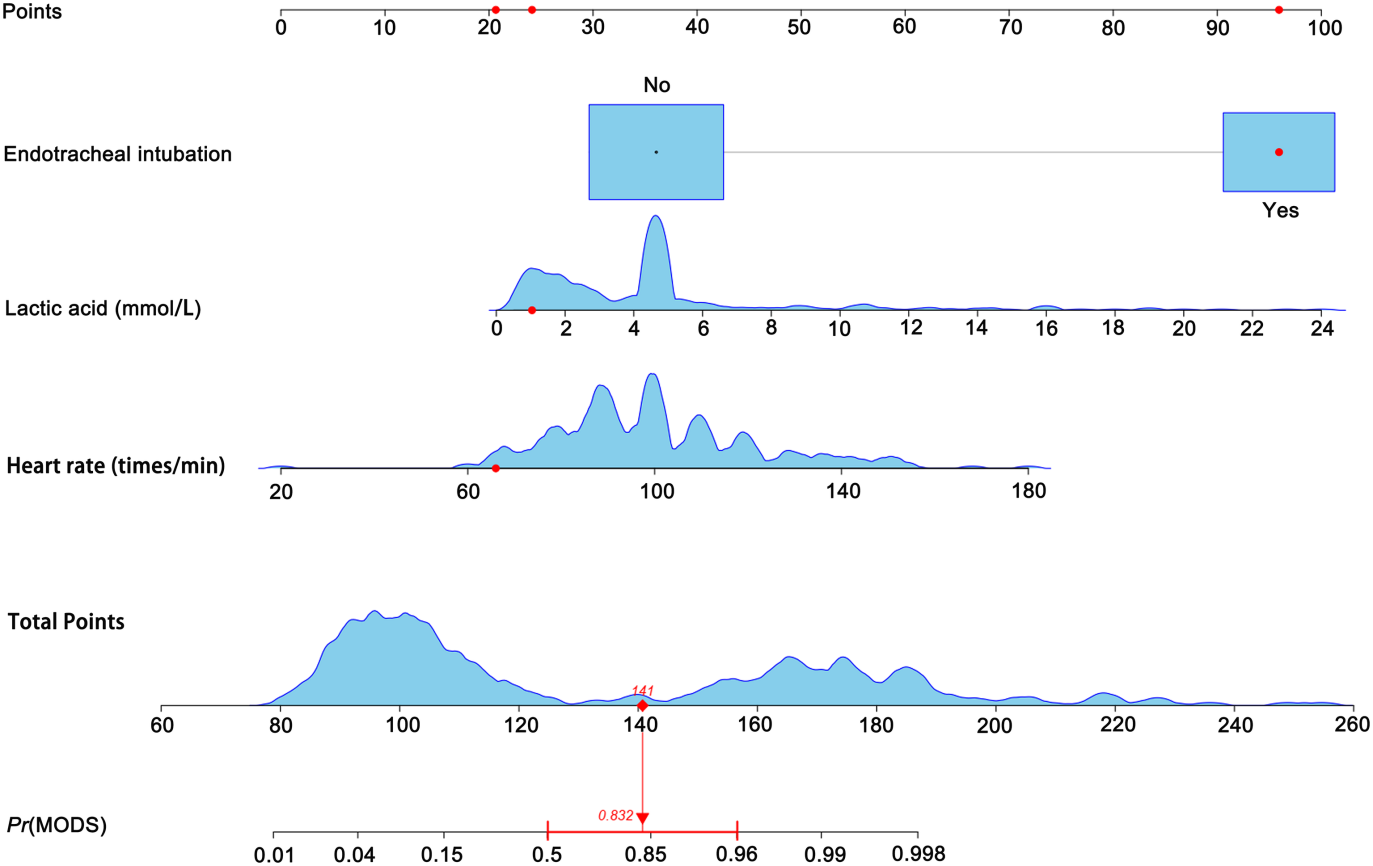
Nomogram for predicting the probability of MODS occurrence in AOPP patients. As shown in the figure, a patient included in this study achieved a total score of 141, with a corresponding MODS predicted probability of 0.832 (83.2%).

**Figure 3:**
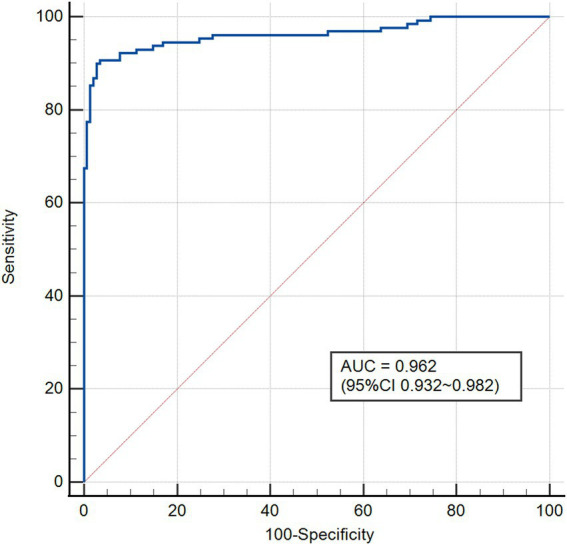
ROC curve of the predictive model.

Based on the aforementioned three predictors, we constructed a logistic regression risk prediction model for forecasting MODS occurrence in AOPP patients and developed a nomogram. The nomogram uses the real-time measured heart rate and lactate to calculate the corresponding score, which can provide a more precise and individualized prediction of MODS risk. As shown in Figure 2, a patient achieved a total score of 141, with a corresponding MODS predicted probability of 0.832 (83.2%). The final model yielded a C-index of 0.962 (95% CI 0.932–0.982), as shown in Figure 3.

### 3.5 Model performance evaluation

**Figure 4:**
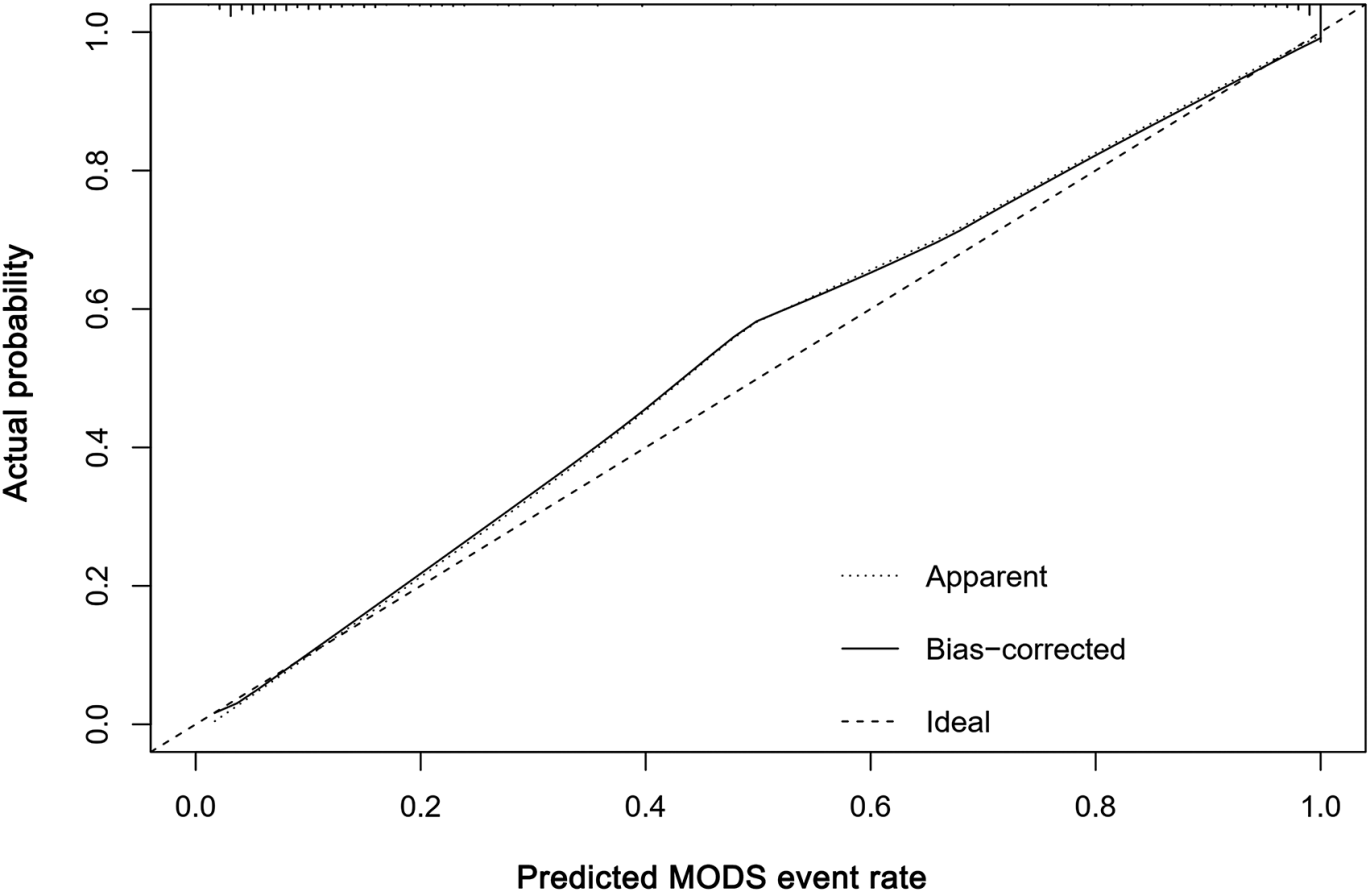
Calibration curve based on the predictive model.

**Figure 5:**
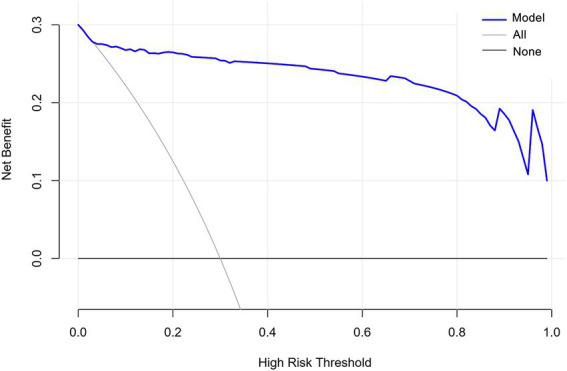
Clinical decision curve based on predictive models.

The nomogram calibration curve indicates favorable predictive outcomes (Figure 4). As illustrated in Figure 5, DCA analysis indicates that this nomogram demonstrates a significant overall net benefit advantage across a broad range of practical threshold probabilities, revealing substantial clinical application potential. As shown in Table 4, internal Bootstrap validation confirmed the robustness of the model, with a validated C-index of 0.959, consistent with the original model.

**Table 4:** Bootstrap internal validation analysis of MODS occurrence in AOPP patients.

Variable	Predictive model (OR (95% CI))	Bootstrap (OR (95% CI))^a^
Heart rate (times/min)	1.044 (1.012 ~ 1.078)	1.046 (1.017 ~ 1.076)
Lactic acid (mmol/L)	1.491 (1.077 ~ 2.065)	1.391 (1.121 ~ 1.728)
Endotracheal intubation [*n*(%)]	165.336 (29.966 ~ 912.240)	397.405 (89.260 ~ 1769.332)
C index	0.962	0.959

a Bootstrap internal validation was performed to evaluate model stability.

AOPP, acute organophosphorus pesticide poisoning; MODS, multiple organ dysfunction syndrome; OR, odds ratio; CI, confidence interval.

## 4 Discussion

Acute organophosphorus pesticide poisoning is one of the most common acute toxic emergencies worldwide, especially in developing agricultural countries and regions (1). Multiple organ dysfunction syndrome is the most serious complication and the leading cause of death in severe AOPP patients (18). Previous studies have reported that the incidence of MODS in AOPP patients ranges from 30 to 50%, and the mortality rate of critically ill patients exceeds 30% (10). Early and accurate risk stratification is crucial to improve patient prognosis. This study developed and internally validated a clinical nomogram model for predicting MODS in AOPP patients based on three easily available indicators: heart rate, endotracheal intubation and blood lactic acid. The model showed excellent discriminative efficacy (C-index 0.962), good calibration degree and favorable clinical net benefit in a wide range of threshold probabilities. To date, this study is one of the few special prediction models for MODS in AOPP patients constructed by combining vital signs, treatment intervention indicators and biochemical markers, providing a quantitative and visualized bedside risk assessment tool for clinical practice.

Previous studies on prognostic evaluation of AOPP have mostly focused on predicting overall mortality, while special prediction models for MODS are relatively limited (19). Some studies have used single biomarkers such as cholinesterase, creatinine or lactic acid to evaluate the severity of poisoning, but single indicators are vulnerable to interference and have limited predictive accuracy (20–22). For example, traditional cholinesterase activity can reflect the degree of enzyme inhibition, but cannot fully represent tissue hypoperfusion, systemic inflammatory response and circulatory disorder, which are the core links leading to MODS. In contrast, the model constructed in this study integrates three complementary dimensions: hemodynamic stress state (heart rate), critical illness intervention measure (endotracheal intubation) and tissue hypoxia degree (blood lactic acid), forming a comprehensive early warning system, which is more clinically practical than single-factor evaluation.

Heart rate was confirmed as an independent predictor in this study, which is consistent with the results of previous related studies (23, 24). Relevant studies have shown that tachycardia in AOPP patients reflects excessive activation of sympathetic-adrenal medulla system, increased myocardial oxygen consumption and systemic stress state, which are closely related to adverse clinical outcomes (25). However, most previous studies only took heart rate as a single descriptive index, and rarely incorporated it into a quantitative prediction model. It should be noted that tachycardia observed in AOPP patients may not only be related to the toxic effects of organophosphorus pesticides but can also be caused iatrogenically by atropine treatment, which is a routine antidote used in clinical practice. Atropine can significantly increase heart rate by blocking muscarinic receptors, which may partially contribute to the elevated heart rate in this study. Therefore, the heart rate value should be interpreted cautiously, taking into account the potential influence of atropine therapy during clinical application of the nomogram.

Endotracheal intubation is the strongest predictor in this model, with an extremely high odds ratio. This result is consistent with previous studies that mechanical ventilation demand indicates severe respiratory failure, airway protection function loss and severe central nervous system depression, suggesting a high risk of subsequent MODS (26, 27). Although mechanical ventilation is a life-saving treatment, it may also induce ventilator-associated lung injury, systemic inflammatory response and secondary organ dysfunction (27, 28). Notably, few existing AOPP prediction models take endotracheal intubation as a core variable (29). The results of this study highlight that the intervention status itself can be used as a highly effective predictive marker, which is easy to obtain in emergency settings.

Blood lactic acid was confirmed as an independent risk factor for MODS, which is consistent with the evidence of critical care medicine and poisoning research (30). Lactate is a sensitive indicator reflecting tissue hypoperfusion and cellular hypoxia, which is the core pathophysiological link of MODS induced by AOPP (31, 32). Previous studies have confirmed that elevated blood lactic acid is related to the mortality of AOPP patients (1, 18, 33), but few studies have specifically verified its value in predicting MODS. This study further confirmed that lactic acid can provide independent prognostic information beyond vital signs and intervention measures, and dynamic monitoring of lactic acid helps to identify patients at high risk of multiple organ injury before obvious organ dysfunction.

Compared with similar AOPP prognosis models, the nomogram constructed in this study has obvious advantages. First, the model takes MODS as the prediction endpoint, not general mortality, which makes up for the blank of clinical application. Second, only three routine available indicators are used, which is simple and fast, and is suitable for primary hospitals with limited medical resources. Third, the model has excellent discriminative ability (AUC = 0.962), which is higher than most reported AOPP scoring systems or single biomarker detection methods. Fourth, internal Bootstrap verification confirms the robustness of the model, which provides a reliability basis for further external verification and clinical application.

This study also has some limitations. First, this is a single-center retrospective study, which may have selection bias. Second, the sample size is limited, and only internal verification is carried out. It is necessary to carry out external verification in multiple medical centers before large-scale clinical application. Third, some key factors such as pesticide type, poisoning dose, treatment time and specific antidote use scheme are not included in the analysis. In the future, multi-center prospective research design can be adopted, integrate more pathogenic and treatment-related variables, and carry out long-term prognosis follow-up to further optimize and improve the model.

In conclusion, this study constructed a concise and high-precision nomogram model for predicting MODS in AOPP patients based on heart rate, endotracheal intubation and blood lactic acid. This model is superior to many previous single-indicator or non-MODS-specific prediction tools, and provides a visualized and quantitative method for early risk stratification. After external verification, the model can help clinicians identify high-risk patients at an early stage, rationally allocate critical care resources, carry out targeted intervention measures, reduce the incidence of MODS, and ultimately improve the prognosis of AOPP patients.

## Data Availability

The raw data supporting the conclusions of this article will be made available by the authors, without undue reservation.
